# Factors associated with 5-min APGAR score, death and survival in neonatal intensive care: a case-control study

**DOI:** 10.1186/s12887-022-03592-9

**Published:** 2022-09-23

**Authors:** Victória Brioso Tavares, Josiel de Souza e Souza, Márcio Vinicius de Gouveia Affonso, Emerson Souza Da Rocha, Lucio Flavio Garcia Rodrigues, Luciana de Fátima da Costa Moraes, Gabrielly Cristiny dos Santos Coelho, Sabrina Souza Araújo, Pablo Fabiano Moura das Neves, Fabiana de Campos Gomes, João Simão de Melo-Neto

**Affiliations:** 1 Federal University of Pará (UFPA), Belém, PA Brazil; 2 State University of Pará (UEPA), Belém, PA Brazil; 3 Ceres Faculty of Medicine (FACERES), São José do Rio Preto, SP Brazil; 4 Clinical and Experimental Research Unit of the Urogenital System (UPCEURG), Institute of Health Sciences of the Federal University of Pará. João de Barros Barreto Hospital, Mundurucus street, 4487; Guamá, Belém, PA CEP: 66073-000 Brazil

**Keywords:** Apgar score, Child health services, Intensive care units, neonatal

## Abstract

**Background:**

The 5-minute APGAR score is clinically used as a screening tool to assess how the newborn has reacted to previous care, remaining relevant for predicting neonatal survival. This study aimed to analyze the determinants of the 5th minute APGAR score, and the factors associated with the death and survival of newborns with low APGAR scores hospitalized in the neonatal intensive care unit (NICU) at a referral public hospital in North Brazil.

**Methods:**

This was a hospital-based retrospective case-control study with 277 medical records. Newborns who presented with a 1-minute APGAR score < 7 followed by a 5-minute APGAR score < 7 were considered cases, while a score ≥ 7 was categorized as controls. Univariate and multivariable logistic regression analyses were used to establish the determinant factors of the low APGAR score and death outcome in this group. Survival curves were obtained using the Kaplan-Meier estimator, and then univariate and multivariate Cox regression was performed.

**Results:**

After adjusted analysis, the factor associated with low APGAR scores was vaginal delivery (OR = 3.25, 95%CI = 1.60–6.62, *p* = 0.001). Birth injury (OR = 0.39, 95%CI = 0.19–0.83, *p* = 0.014) was associated with upper APGAR scores. No significant independent associations were observed between the variables analyzed and death in the low APGAR score group. The Kaplan-Meier curve showed that individuals who presented Cesarean delivery had a shorter survival time in the ICU.

**Conclusion:**

In this setting, a 5-minute Apgar score < 7 was associated with the occurrence of vaginal delivery and birth injury with a 5-minute Apgar score ≥ 7. Survival in ICU was lower in newborns that were delivered via cesarean section.

## Introduction

In Brazil, 303,260 neonatal deaths were recorded from 2007 to 2017, with an average Neonatal Mortality Rate (NMR) of 9.46 per 1000 live births [1]. The country achieved a considerable reduction in the NMR, from 25.33/1000 in 1990 to 8.5/1000 live births in 2019, although it has recently shown a significant deceleration [1, 2]. The improvement of socioeconomic and health policies, such as the implementation of the Unified Health System (SUS) [3]; the “Family Health Strategy” [4], the “*Rede Cegonha”* strategy, and the “*Bolsa Família Program”* [5, 6], positively impacted the reduction of mortality rates. Still, the infant and child mortality indicators remain high by international standards, with a concentration of deaths in less developed regions, especially the Northern region of Brazil, which has the highest neonatal mortality rate with 11.02 per 1000 live births [1, 7–10].

Prematurity is one of the most significant factors contributing to the high rate of neonatal mortality, representing approximately 7.2% of deaths in live births [11, 12], with congenital abnormalities, perinatal asphyxia, birth injury, and lower respiratory tract infections also being frequent causes [13–15]. In the northern region, the highest proportion of registered deaths was due to infection (26.9%), followed by prematurity, congenital malformation, and perinatal asphyxia. It should be noted that early NMR associated with perinatal asphyxia in Brazil is still high even in neonates with proper birth weight and without congenital malformations [16, 17].

Prematurity and respiratory problems accounted for the greatest increases in hospitalizations among neonates in the neonatal intensive care unit (NICU), mainly due to inefficient maturity of the respiratory system and greater susceptibility to infection [18–20]. In that regard, vaginal delivery accounted for the most vulnerable group to perinatal asphyxia, indicating the importance of avoiding the pilgrimage of women to maternity hospitals, highly skilled personnel trained for resuscitation in the hospital, and greatest intrapartum care (assisted care) [17].

A low Apgar score is an important factor correlated with increased mortality in term and preterm infants without congenital anomalies [21]. It is clinically used to assess hemodynamic impairment such as apnea, bradycardia, cyanosis, hypoperfusion, hypotonia, or respiratory depression. Is used clinically in the first minute as a screening tool to assess the need for early intervention, and in the fifth minute to assess how the newborn reacted to previous care. Still, in the fifth minute, the Apgar score is also relevant for the prediction of neonatal survival [22, 23]. Thus, a cutoff value of 7 points for low and high scores has been discussed by different studies and neurologists [24–27].

Identifying factors associated with the 5-minute Apgar score and death could be helpful in building strategies to reduce the number of neonatal deaths and morbidity associated with a low Apgar score. Therefore, this study aimed to analyze the determinants of the 5-minute Apgar score and determine the factors associated with death and survival in newborns with low Apgar scores who were hospitalized in the NICU at a referral public hospital in Northern Brazil.

## Methods

### Study design

This was a hospital-based, unmatched, observational, and retrospective case-control study with descriptive and inferential analyses.

### Setting and period of study

The study was conducted using data from medical records of patients treated at a maternal and child health referral hospital in the northern region of Brazil, located in the municipality of Belém (PA), in 2017.

This study took place at a public tertiary-level reference hospital providing maternal-infant care aligned with the Brazilian National Humanization Policy, which aims to implement the SUS principles in daily care and management practices, qualify public health, and encourage solidarity exchanges between managers, workers, and users; the hospital also provided care in line with the *Rede Cegonha* strategy that encourages humanized care during pregnancy, childbirth, and the puerperium [28]. In addition, the hospital adopts the internal policy of promoting assisted vaginal delivery as it is considered an indicator of the quality of service and referring neonates with a low Apgar score in the 1st minute directly to the NICU where they are monitored, and anthropometrical and postnatal data are recorded according to a specific hospital data instrument.

In the year of the study, 10,460 deliveries were performed, with 5263 normal deliveries (50.4%) and 5197 (49.6%) cesarean sections. A neonatal mortality rate of 12,8% was reported. Regarding physical and professional infrastructure, the hospital had 486 beds, 60 of which were exclusive to the NICU, and a total of 2573 servers, with a total of 122 servers in the delivery room (25 pediatricians, 6 gynecologists/obstetricians, 27 nurses, and 51 nursing technicians). The hospital had a general protocol for cardiopulmonary resuscitation, with 565 service employees trained at the time of the study [29], of with 34 delivery room employees were trained by the Neonatal Resuscitation Programme of the Brazilian Pediatric Society in the year of study (21 pediatricians, and 13 nurses).

### Population

The study was conducted with neonates of both sexes admitted to the NICU of the referral hospital.

### Eligibility criteria

The medical records of neonates who presented with a 1-minute Apgar score < 7 and were referred to the NICU were included, and so the 5-minute Apgar score was assessed.

Of the deliveries performed in 2017, stillbirths, deaths in the delivery room, and other neonates who were not referred to the neonatal ICU were excluded. Incomplete medical records on Apgar scores, patients with major congenital anomalies (such as anencephaly, severe hydrocephalus, and gastroschisis), and newborns of mothers who received general anesthesia were excluded due to the neonate’s increased chances of receiving a low Apgar score.

### Sampling

Probabilistic random sampling was used to select patients.

### Sample

The sample size calculation considered 80% power of the study, based on a variable determined randomly by Yeshaneh et al. (2021) [30]. This presented an odds ratio (OR) of 2.3 (1.10–4.71) with a *p*-value < 0.05, indicating a minimum sample size of 242 participants, with a minimum of 81 individuals in group 1 (Apgar Score < 7) and 161 in group 2 (Apgar Score ≥ 7). This was based on the N2 / N1 allocation ratio (0.502) and proportion p2 or control-to-case ratio (0.155), with an α and β error of 0.05 and 0.2, respectively.

**Fig. 1:**
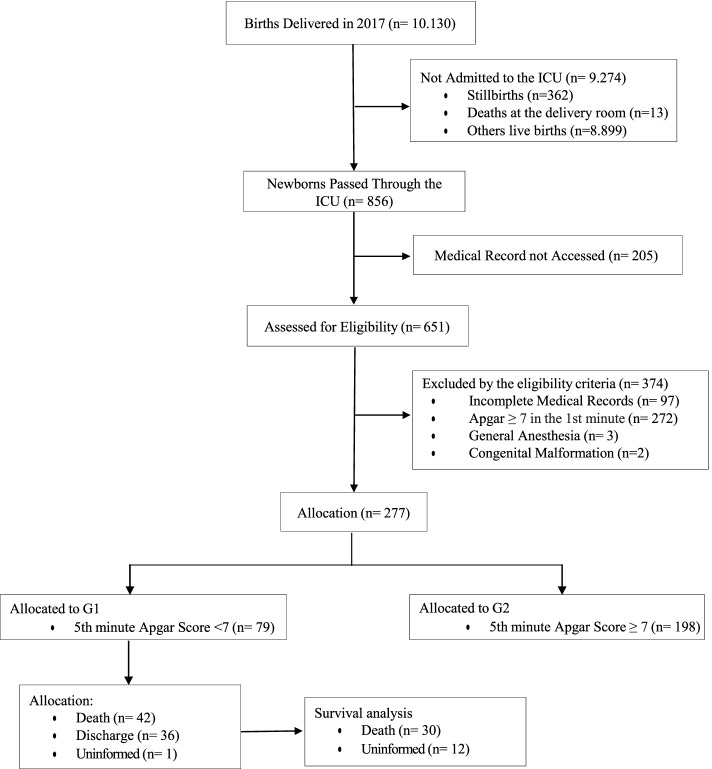
Flowchart of the selection and distribution of individuals in the groups

The initial sample consisted of 651 medical records, of which 277 were selected after the eligibility criteria. Newborns with a 5-minute Apgar score < 7 were categorized as cases (G1), and newborns with a 5-minute Apgar score ≥ 7 were categorized as controls (G2) (Fig. 1).

### Data collection and variables

Data were collected by reviewing medical records. The Apgar score was estimated using five items, ​​and the final score was calculated from the sum of each item [22]. A form developed by the authors was used for data collection. The following data were included: (1) maternal factors: maternal age, number, gestation, deliveries, and abortions; gestation time (extremely preterm: < 28 weeks, very preterm: 28 to 31 weeks, moderate and late preterm: 32 to 36 weeks, term: 37–41 weeks, post-term: 42 weeks or more) [31, 32]; occurrence and number of prenatal care; gestational complications (leukorrhea, hypertensive disorders of pregnancy [HDP], preeclampsia, anemia, bleeding, and urinary tract infections [UTIs]); (2) obstetric conditions: type of delivery (cesarean or vaginal), single birth, and birth injury, defined as “*the structural destruction or functional deterioration of the neonate’s body due to a traumatic event at birth*” by Akangire and Carter [33] in 2016; (3) anthropometric data: height, weight, cephalic perimeter, thoracic perimeter, abdominal perimeter, and sex; and (4) clinical postnatal data: ventilatory support (continuous positive airway pressure [CPAP], invasive mechanical ventilation [IMV], and surfactant supplementation) and outcomes (discharge or death).

### Primary outcomes

The primary outcome considered the association between maternal characteristics and obstetric conditions as determinants of the 5-minute Apgar score < 7.

### Statistical analysis

Descriptive statistical analysis was conducted to compute frequency (absolute and relative), mean, and standard deviation (parametric) or medians with interquartile range (IQR, non-parametric) for each group.

Binary logistic regression analysis was used to establish the determinant factors of the low Apgar score and death outcome in the low Apgar score cohort. Initially, we performed a univariate analysis, considering a *p*-value < 0.25 [30]. To verify the multicollinearity, the Variance Inflation Factor (VIF) was calculated and the variables that presented a VIF value above 10 were removed from the final model. Statistical significance was set at *p* < 0.05. OR with 95% confidence intervals (95% CI) were used to quantify the degree of association.

Survival curves were obtained using the Kaplan-Meier estimator, and log-rank (initial), Breslow (intermediary), and Tarone-Ware (final) tests were used to identify the occurrence of a statistically significant difference in the different periods [34].

## Results

### Factors associated with the 5-minute Apgar score

Seventy-nine individuals were included in G1 and 198 in G2, according to the eligibility criteria (Fig. 1).

Table 1 presents the analysis of the variables (maternal, obstetric and postnatal characteristics) and conditions for both groups.

**Table 1:** Analyses of maternal, obstetrical, postnatal and anthropometric characteristics and conditions for 5th minute Apgar Score groups

Characteristic	Overall	G1 5 minute Apgar Score < 7 ***N*** = 79^**a**^	G2 5 minute Apgar Score ≥ 7 ***N*** = 198^**a**^	***p*** value
**Maternal characteristics**				
**Number of deliveries**	1 (0, 2)	1 (0, 1)	1 (0, 2)	0.035*
**Gestation time in weeks**				0.003*
< 28	42 (15.4%)	20 (25.6%)^b^	22 (11.3%)^b^	
28–31	55 (20.1%)	11 (14.1%)	44 (22.6%)	
32–36	120 (44%)	26 (33.3%)^b^	94 (48.2%)^b^	
37–41	53 (19.4%)	19 (24.4%)	34 (17.4%)	
≥ 42	3 (1.1%)	2 (2.6%)	1 (0.5%)	
**Obstetrical conditions**				
**Type of delivery**				< 0.001*
Cesarean	160 (58%)	28 (35%)	132 (67%)	
Vaginal	117 (42%)	51 (65%)	66 (33%)	
**Birth injury**	70 (27%)	31 (41%)	39 (22%)	0.001*
**Postnatal conditions**				
**Surfactant**	116 (43%)	41 (53%)	75 (39%)	0.039*
**IMV**	110 (41%)	45 (58%)	65 (34%)	< 0.001*
**CPAP**	41 (15%)	6 (7.7%)	35 (18%)	0.030*
**Outcome**				< 0.001*
Discharge	168 (62%)	36 (46%)	132 (68%)	
Death	105 (38%)	42 (54%)	63 (32%)	

*IMV* invasive mechanical ventilation, *CPAP* continuous positive airway pressure, *OR* Odds Ratio, *CI* Confidence Interval

^*^p-value ≤ 0.05

^a^Median (IQR); n (%)

^b^Adjusted residual post-hoc tests with Z_crit_ = 1.96

The overall median number of deliveries was 1, with G1 presenting a lower IQR (*p* = 0.035) (Table 1). Gestation time showed that neonates with ≤28 weeks of gestation had worse Apgar scores than G2 (G1 = 25.6% vs. G2 = 11.3%) (Table 1). Conversely, neonates with a gestational age between 32 and 36 weeks had better scores concerning the proportion of neonates (*p* = 0.003) (Table 1).

The other obstetric variables were not significant: mothers’ age (years) (G1: 25 (20, 30), G2: 25 (20, 31); *p* = 0.700), number of gestations (G1: 2 (1, 3), G2: 2 (1, 3); *p* = 0.076), number of abortions (G1: 0 (0, 0), G2: 0 (0, 0); *p* = 0.600), gestation time (continuous) (G1: 34 (26, 37), G2: 33 (30, 35); *p* > 0.900), prenatal care (G1: 61 (79%), G2: 169 (87%); *p* = 0.100), number of prenatal care (G1: 2 (1, 4), G2: 3 (2, 4); *p* = 0.150), UTIs (G1: 28 (35%), G2: 75 (38%); *p* = 0.700), leukorrhea (G1: 28 (35%), G2: 66 (33%); *p* = 0.700), anemia (G1: 3 (4.1%), G2: 22 (12%); *p* = 0.056), bleeding (G1: 7 (9.5%), G2:33 (18%); p = 0.100), preeclampsia (G1: 3 (4.1%), G2: 19 (10%); *p* = 0.110), HDP (G1: 10 (14%), G2: 29 (16%); *p* = 0.700).

Cesarean was the overall most frequent type of delivery (58%), although vaginal delivery was the most present in G1 (65%, *p* < 0.001), birth injury (41%, *p* = 0.001), IMV (58%, *p* < 0.001), and death (54%, *p* < 0.001) were also most common in G1 (Table 1). Surfactant supplementation (53%, *p* = 0.039) were most common in G1 and CPAP (18%, *p* = 0.030) in G2 (Table 1). Regarding obstetric conditions, there was no significant difference only for single birth (G1: 68 (86%), G2: 172 (88%); *p* = 0.700).

Anthropometric characteristics showed no significant differences: weight (g) (G1: 1.612 (899, 2.348), G2: 1.415 (950, 2.075); *p* = 0.400), height (cm) (G1: 24.0 (11.0, 36.0), G2: 24.0 (14.0, 35.0); *p* > 0.900), cephalic (cm) (G1: 20.0 (10.0, 27.0), G2: 19.0 (11.0, 25.0); *p* = 0.700) and thoracic (cm) (G1: 18.0 (10.0, 26.0), G2: 16.0 (11.0, 24.0); *p* = 0.500), abdominal perimeter (cm) (G1: 19.0 (11.0, 26.0), G2: 17.0 (11.0, 24.0); *p* = 0.500). In addition, sex (Female G1: 31 (40%), G2: 89 (46%); Male G1: 47 (60%), G2: 104 (54%); *p* = 0.300) also showed no difference between groups.

Table 2 shows the results of the univariate and multivariate logistic regression analyses. In the univariate analysis, the following variables did not have a *p*-value to be included in the final model: mothers’ age (years) (OR = 1.00, 95%CI: 0.96–1.03, *p* = 0.80), number of abortions (OR = 1.11, 95%CI: 0.73–1.63, *p* = 0.62), gestation time (continuous) (OR = 0.99, 95%CI: 0.93–1.05, *p* = 0.67), UTIs (OR = 0.90, 95%CI: 0.52–1.54, p = 0.70), leukorrhea (OR = 1.10, 95%CI: 0.63–2.89, *p* = 0.73), HDP (OR = 0.85, 95%CI: 0.38–1.80, *p* = 0.68) and single birth (OR = 0.86, 95%CI: 0.41–1.92, *p* = 0.708). Univariate analysis showed a *p* < 0.25 for the following variables: number of deliveries, gestation time, prenatal care, number of prenatal care, anemia, bleeding, preeclampsia, vaginal delivery, and birth injury (Table 2). There was no multicollinearity between the variables with *p*-values < 0.25, therefore, all variables were included. Multivariate analysis showed that vaginal delivery (OR = 3.25, 95%CI = 1.60–6.62, *p* = 0.001) was associated with a 5-minute Apgar score < 7 and birth injury (OR = 0.39, 95%CI = 0.19–0.83, *p* = 0.014) was associated with > 7.

**Table 2:** Univariate and multivariate analyses of maternal and obstetrical characteristics and conditions of newborns with 5 minute Apgar Score < 7

Characteristic	N	OR^**1**^	95% CI^**1**^	***p***-value^**1**^	N	OR^**2**^	95% CI^**2**^	***p***-value^**2**^
**Maternal characteristics**								
**Number of deliveries**	273	0.80	0.65, 0.97	0.020^**#**^	207	1.12	0.88, 1.42	0.354
**Gestation time in weeks**	273			< 0.0001^**#**^	207			0.096
< 28		–	–			–	–	
28–31		3.64	1.48, 8.91			3.57	1.10, 11.58^a^	
32–36		3.29	1.56, 6.92			1.83	0.67, 5.01	
37–41		1.63	0.71, 3.71			0.56	0.03, 9.94	
≥ 42		0.45	0.04, 5.40			0.95	0.29, 3.06	
**Prenatal care**	271	0.56	0.28, 1.14	0.11^**#**^	207	1.26	0.42, 3.79	0.670
**Number of prenatal care**	250	0.93	0.82, 1.05	0.22^**#**^	207	0.99	0.83, 1.19	0.960
**Anemia**	261	0.32	0.07, 0.95	0.04^**#**^	207	2.57	0.64, 10.29	0.182
**Bleeding**	261	0.49	0.19, 1.10	0.08^**#**^	207	2.70	0.91, 8.02	0.074
**Preeclampsia**	261	0.37	0.09, 1.14	0.08^**#**^	207	1.44	0.33, 6.29	0.630
**Obstetrical conditions**								
**Type of delivery (Vaginal)**	277	3.64	2.12, 6.36	< 0.001^**#**^	207	3.25	1.60, 6.62^a^	0.001*
**Birth Injury**	256	2.57	1.43, 4.59	0.002^**#**^	207	0.39	0.19, 0.83^a^	0.014*

^1^*OR* Odds Ratio, *CI* Confidence Interval

^2^*aOR* Adjusted Odds Ratio, *CI* Confidence Interval

^**#**^*p*-value < 0.25

^*^p-value ≤0.05

^a^IC95% significant

### Factors associated with mortality and discharge in newborns with a low 5-minute Apgar score

The analysis included 79 newborns with a 5-minute APGAR score < 7, with 42 cases of death, 36 cases of discharge, and 1 case discarded due to missing data (Fig. 1).

Table 3 presents the analysis of each variable category. The overall median number of abortions was 0, with neonates who were discharged presenting a higher IQR (median = 0, IQR = 0–1, *p* = 0.012) than neonates who died. Gestation time was significantly different between the groups, with death in 37% of neonates with a gestation time between < 28 weeks, and a higher number of discharges between 37 and 41 weeks (*p* = 0.021).

**Table 3:** Descriptive analyses of maternal, obstetrical, postnatal and anthropometric characteristics and conditions for discharge and death outcome of newborns with 5 minute Apgar Score < 7

Characteristic	Overall	Death ***N*** = 42^**a**^	Discharge ***N*** = 36^**a**^	***p***-value
**Maternal characteristics**				
**Number of abortions**	0 (0, 0)	0 (0, 0)	0 (0, 1)	0.012*
**Gestation time (continuous)**	34 (28, 37)	35 (32, 37)	30 (26, 35)	0.003*
**Gestation time in weeks**				0.021*
< 28	19 (24.7%)	15 (36.6%)^b^	4 (11.1%)^b^	
28–31	11 (14.3%)	8 (19.5%)	3 (8.3%)	
32–36	26 (33.8%)	11 (26.8%)	15 (41.7%)	
37–41	19 (24.7%)	6 (14.6%)^b^	13 (36.1%)^b^	
≥42	2 (2.6%)	1 (2.4%)	1 (2.8%)	
**Obstetrical conditions**				
**Birth injury**	31 (42%)	8 (24%)	23 (56%)	0.006*
**Postnatal conditions**				
**Surfactant**	41 (53%)	14 (40%)	27 (64%)	0.033*
**IMV**	45 (58%)	16 (46%)	29 (69%)	0.039*
**CPAP**	6 (7.8%)	6 (17%)	0 (0%)	0.007*
**Anthropometric characteristics**				
**Weight (g)**	1.635 (955, 2.350)	2.045 (1.436, 2.819)	1.198 (711, 1.970)	< 0.001*
**Height (cm)**	24 (12, 36)	33 (22, 38)	18 (10, 30)	< 0.001*
**Cephalic (cm)**	20 (11, 27)	24 (18, 28)	12 (7, 23)	< 0.001*
**Toracic (cm)**	18 (11, 26)	22 (18, 28)	13 (8, 23)	< 0.001*
**Abdominal (cm)**	19 (11, 26)	25 (18, 31)	21 (8, 22)	< 0.001*

*IMV* invasive mechanical ventilation, *CPAP* continuous positive airway pressure, *OR* Odds Ratio, *CI* Confidence Interval

^*^p-value ≤ 0.05

^a^Median (IQR); n (%)

^b^Adjusted residual post-hoc tests with Z_crit_ = 1.96

Birth injury (56%, *p* = 0.006), surfactant supplementation (64%, *p* = 0.033), and IMV (69%, *p* = 0.039) were most frequent in neonates who were discharged. Weight (g) (median = 2.045, 95%CI = 1.436–2.819), height (cm) (median = 45.0, 95%CI = 39.0–48.2), and cephalic (median = 31.8, 95%CI = 28.4–33.6), thoracic (median = 27.0, 95%CI = 24.9–30.0), abdominal (median = 26.5, 95%CI = 23.2–29.8) perimeters and CPAP (17%, *p* = 0.007) were higher in neonates who died.

The other variables did not show statistically significant differences between the groups. The following maternal characteristics showed no differences: mothers’ age (years) (Death: 26 (20, 31); Discharge: 24 (20, 28); *p* = 0.300), number of gestations (Death: 1 (1, 4); Discharge: 2 (1, 3); *p* > 0.900), number of deliveries (Death: 1 (0, 2); Discharge: 1 (0, 1); *p* = 0.110), number of prenatal care (Death: 3 (1, 5); Discharge: 2 (1, 3); *p* = 0.100), UTIs (Death: 15 (42%); Discharge: 13 (31%); *p* = 0.300), leukorrhea (Death: 12 (33%); Discharge: 15 (36%); *p* = 0.800), Anemia (Death: 1 (3.0%); Discharge: 2 (5.0%); p > 0.900), Bleeding (Death: 1 (3.0%); Discharge: 6 (15%); *p* = 0.120), Preeclampsia (Death: 3 (9.1%); Discharge: 0 (0%); *p* = 0.088), HDP (Death: 6 (18%); Discharge: 4 (10%); p = 0.300). The following obstetrical conditions: type of delivery (Cesarean - Death: 11 (26%); Discharge: 17 (47%); Vaginal - Death: 31 (74%); Discharge: 19 (53%); *p* = 0.054), Single birth (Death: 32 (89%); Discharge: 35 (83%); *p* = 0.500) showed no differences. Finally, the sex variable (female - Death: 16 (44%), Discharge:14 (34%); Male - Death: 20 (56%); Discharge: 27 (66%); *p* = 0.400) there was no difference between the outcomes.

Table 4 presents the univariate and multivariable logistic regression analysis. In the univariate analysis, the following variables did not have a *p*-value to be included in the final model: mothers’ age (years) (OR = 0.97, 95%CI: 0.91–1.03, *p* = 0.318), number of gestation (OR = 0.96, 95%CI: 0.70–1.31, *p* = 0.775), prenatal care (OR = 1.35, 95%CI: 0.43–4.29, *p* = 0.606), UTI (OR = 0.63, 95%CI: 0.24–1.59, *p* = 0.326), leukorrhea (OR = 1.11, 95%CI: 0.44, 2.87, *p* = 0.826), anemia (OR = 1.68, 95%CI: 0.15–37.2, *p* = 0.669), HDP (OR = 0.50, 95%CI: 0.12–1.92, *p* = 0.312), Single birth (OR = 0.63, 95%CI: 0.15–2.27, *p* = 0.479), sex (Male) (OR = 1.54, 95%CI: 0.62, 3.92, *p* = 0.355). Univariate analysis showed a *p* < 0.25 for the following variables: number of deliveries, number of abortions, gestation time (continuous and in weeks), number of prenatal care, bleeding, preeclampsia, type of delivery (vaginal), birth injury, surfactant supplementation, IMV, CPAP, weight, height, cephalic, thoracic and abdominal perimeters. The final model of the multivariable logistic regression didn’t include the variables weight, gestation time (continuous and in weeks), thoracic and abdominal perimeter, due to multicollinearity (VIF > 10). No significant independent associations were observed in multivariable analysis.

**Table 4:** Univariate and multivariate analyses of maternal, obstetrical, postnatal and anthropometric characteristics and conditions for discharge and death outcome of newborns with 5 minute Apgar Score < 7

Characteristic	N	OR^**1**^	95% CI^**1**^	***p***-value^**1**^	N	OR^**2**^	95% CI^**2**^	***p***-value^**2**^
**Obstetrical conditions**								
**Number of deliveries**	76	0.68	0.44, 1.00	0.051^**#**^	60	0.65	0.37, 1.17	0.154
**Number of abortion**	76	2.52	1.12, 7.31	0.024^**#**^	60	3.21	0.78, 13.11	0.104
**Gestation time (continuous)**	77	0.87	0.78, 0.95	0.002^**#␥**^				
**Gestation time in weeks (reference < 28)**	77			0.031*^␥^				
28–31		0.71	0.13, 3.99					
32–36		0.20	0.05, 0.75^➧^					
37–41		0.12	0.03, 0.05^➧^					
≥ 42		0.27	0.01, 5.27					
**Number of prenatal care**	75	0.81	0.65, 0.99	0.037^**#**^	60	0.97	0.69, 1.36	0.863
**Bleeding**	73	5.65	0.90, 110	0.067^**#**^	60	1.28	0.00, 0.00	0.999
**Preeclampsia**^a^	73			0.027^**#**^	60	0.00	0.00, 0.00	0.999
**Obstetrical conditions**								
**Type of delivery (Vaginal)**	78	2.52	0.99, 6.67	0.053^**#**^	60	0.65	0.13, 3.13	0.597
**Birth injury**	73	3.99	1.50, 11.4	0.005^**#**^	60	1.62	0.35, 7.35	0.532
**Postnatal conditions**								
**Surfactant**	77	2.70	1.08, 6.96	0.033^**#**^	60	3.07	0.72, 13.4	0.129
**IMV**	77	2.65	1.05, 6.88	0.038^**#**^	60	1.32	0.29, 5.94	0.716
**CPAP**^**a**^	77			0.002^**#**^	60	0.00	0.00, 0.00	0.999
**Anthropometric characteristics**								
**Weight (g)**	77	1.00	1.00, 1.00	< 0.001^**#␥**^				
**Height (cm)**	78	0.88	0.81, 0.95	< 0.001^**#**^	60	9.961	0.84, 1.09	0.530
**Cephalic (cm)**	78	0.80	0.70, 0.90	< 0.001^**#**^	60	0.89	0.71, 1.11	0.319
**Thoracic (cm)**	78	0.80	0.70, 0.90	< 0.001^**#␥**^				
**Abdominal (cm)**	74	0.80	0.70, 0.89	< 0.001^**#␥**^				

*IMV* invasive mechanical ventilation, *CPAP* continuous positive airway pressure

^1^*OR* Odds Ratio, *CI* Confidence Interval

^2^*aOR* Adjusted Odds Ratio, *CI* Confidence Interval

^**#**^*p*-value < 0.25

*p-value ≤0.05

^**␥**^Excluded due multicollinearity analysis

^➧^IC95% significant

^a^OR and aOR value not estimated due to the presence of 0 in one of the categories

### Survival in newborns with a low 5-minute Apgar score

**Fig. 2:**
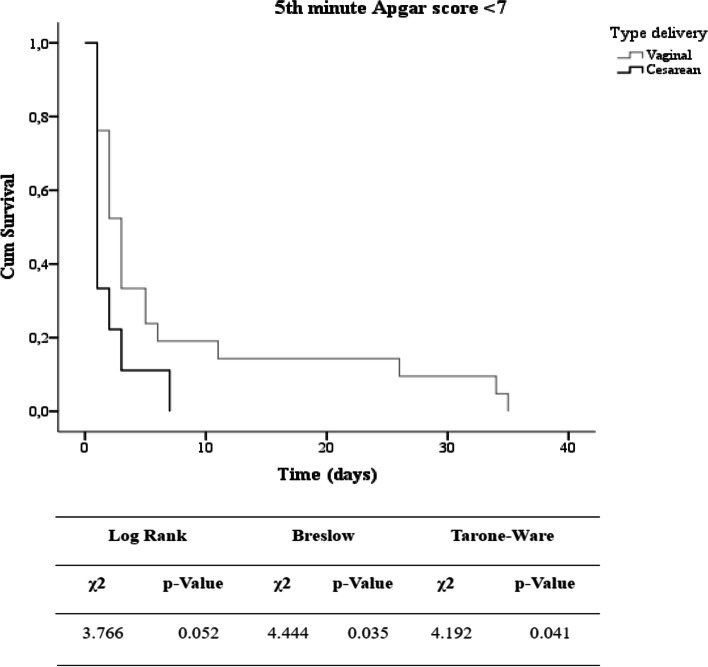
Survival in newborns with low 5-minute Apgar score. Legend: Survival analysis of birth injury and type of delivery variable in newborns with low 5-minute APGAR score

Of the 42 newborns with Apgar < 7 who died, 30 were included in the survival analysis after neonates were excluded because of missing date-of-death data. Figure 2 shows the Kaplan-Meier curve, which indicates that the variable type of cesarean delivery in the middle (*p* = 0.035) and last (*p* = 0.041) part of the curve has a shorter survival time in the NICU. There were no significant differences in the other variables (*p* > 0.05).

## Discussion

Among the factors associated with a 5-minute Apgar score < 7, only vaginal delivery had a threefold association. In addition, the presence of birth injury was associated with an Apgar score ≥ 7.

The relationship between vaginal delivery and an Apgar score < 7 may be due to events and complications during childbirth, such as breech presentation and labor duration, which may affect the choice of the delivery method [21, 35, 36].

Birth injury in literature appeared to be associated with the delivery method, birth weight, maternal age, and neonatal cephalic perimeter [35–38]. Considering that in our study, due to the occurrence of birth injury at higher gestational ages in the neonates with Apgar scores ≥7, it is possible to assume that this group was longer exposed to the possibility of different types of birth injuries, as the most common: scalp injuries, and clavicular fracture [35], which, when not associated with prematurity, did not impact the Apgar score.

It is worth noting that the hospital is a referral hospital for maternal and infant care and has a policy of promoting vaginal delivery, avoiding unnecessary cesarean deliveries, and providing multiprofessional labor management to improve the quality of care.

Prenatal care is essential in minimizing complications during pregnancy and labor since antenatal care improves the prevention, detection, and treatment of risk factors during pregnancy, thereby decreasing the risk of neonatal mortality. Moreover, access to health services, intervention at the right time, and trained professionals in neonatal resuscitation protocols reduce morbidity and mortality in infants [39–41].

The Brazilian Ministry of Health recommends that at least six prenatal care consultations be carried out; however, for prenatal care to be effective, they have also recommended that it is important to assess pregnant women early with adequate frequency and consistency [42]. In this study, even though most of the women in both groups had received prenatal care, none had reached the average number of recommended consultations.

These findings are in agreement with the regional pattern as the overall frequency of adequate prenatal care among women in the northern region is one of the lowest, which directly increases the difficulty in providing medical care at the right time affecting mortality. Poor access to prenatal care is one of the factors, along with poverty and low education status, that leads to a higher risk of neonatal death by perinatal asphyxia, one of the main causes of neonatal deaths in the country [16, 17, 43, 44].

The North region is the most extensive in the country, with a population density slightly above four inhabitants per km^2^ (almost 16 million people) [45], this characteristic promotes difficulties in spatial distribution and transport to specialized health services leading to limitations to access the health system. Regional inequalities regarding access to health services and quality of prenatal care are directly associated with the pilgrimage to a referral service at the right time. Pilgrimage affected more than 20% of women in the northern region [44].

Although national studies have shown that the largest network of obstetric and neonatal care is outsourced by the SUS, it also has described failures of the public system to adequately provide transfer to center-of-excellence hospitals, which are often located in the capitals [46, 47]. This is the scenario of the institution, which receives women from different regions of the state, often in a complex situation, being an “open door” for women with serious pregnancy complications, which contributes to the results, as the pilgrimage was strongly associated with a premature birth initiated by the provider and an Apgar score < 7 in northern Brazil [44].

Considering that 26% of newborns with Apgar < 7 were term and post-term, it is important to assess the quality of perinatal care at the institution. The hospital has a policy of avoid unnecessary cesarean deliveries and provide assisted and multiprofessional labor management to improve the quality of care. Protocols for maternal and infant care are institutionalized in the obstetric emergency department and the neonatal intensive care unit. There are also institutionalized protocols in the delivery room for continuous monitoring during labor according to the risk classification of pregnant women, including use of obstetric Doppler ultrasound. There are also protocols for the use of prenatal steroids in preterm birth, although in the sample of this study, less than 30% of preterm infants received at least one dose of corticosteroids, which demonstrates the importance of monitoring the effective implementation of these protocols in clinical practice.

Regarding infrastructure and staff, the hospital had an annual occupancy rate of 102.6% due to the use of beds improvised and reserved for surgeries. The northern region of Brazil has one of the highest numbers of beds offered by the SUS, with the state of Pará with 2.02 NICU beds (SUS, non-SUS) per 1000 live births [48]. In addition, the hospital had a total of 122 delivery room staff, 27% of whom were trained according to the Neonatal Resuscitation Programme of the Brazilian Pediatric Society, which is still very low considering that the hospital is a reference for high-risk pregnancies. This also reflects a national scenario, where pediatricians were more often in the delivery room, with 94% of them with at least one training course in neonatal resuscitation, and the nursing team not equally trained [49, 50].

In Brazilian state capitals, studies have shown that the main public maternity hospitals have adequate material and human resources in delivery rooms [49]. Nevertheless, in addition to material support, the implementation of adequate monitoring, and training of delivery room staff in resuscitation are crucial to initiate interventions at the right time, as delays can lead to an Apgar score < 7 and neonatal morbidity and mortality even in term neonates [13, 51–53].

The Apgar score contributed to the improvement of perinatal care by converting clinical analyses into quantitative data. Knowing the potential determinants of an Apgar score < 7 is important for the prevention and early identification of risks to decrease morbidity and mortality in newborns.

Considering that this was a retrospective observational study, the quality of the data was subject to the availability and accuracy of the information in the hospital’s original database. Orientation was carried out with the data collectors to ensure the correct use of the hospital’s electronic medical record system, with the head nurse responsible for the sector and researchers responsible for the preparation of the collection instrument used in the study. Nevertheless, our analysis may have been influenced by the loss of information. Therefore, the number of missing observations was added to each variable in the tables.

## Conclusion

In our center’s patient population, we found that a 5-minute Apgar score < 7 was associated with the occurrence of vaginal delivery, and birth injury with a 5-minute Apgar score > 7. No significant independent associations were observed between the factors studied and death in the low Apgar score cohort. In addition, survival in the NICU was lower in newborns who were delivered via cesarean section.

## Limitation of the study

The main limitation is that the results are restricted to the study setting; therefore, caution must be taken with the generalization of these data in other contexts. The lack of access to data regarding fetal presentation, duration of delivery, and instrumental vaginal delivery can be considered a limitation of the analysis. The investigation of the potential interactions of demographic or socioeconomic aspects with the outcomes was beyond the objective of this study but can be considered a limitation since the literature provided callsigns stating a possible relationship between the two. Information on exposure was subject to observation bias.

## Data Availability

The data that support the findings of this study are available from the Fundação Santa Casa de Misericordia do Pará - Brazil, which was used under license for the current study, and so are not publicly available. Data are, however, available from the authors upon reasonable request and with permission of the hospital.
